# Comparative Evaluation of Phenolic Profile, Antioxidative and Cytotoxic Activities of Different *Geranium* Species

**Published:** 2017

**Authors:** Didem Şöhretoğlu, Yasin Genç, Şebnem Harput

**Affiliations:** *Hacettepe University, Faculty of Pharmacy, Department of Pharmacognosy, TR-06100, Ankara, Turkey.*

## Introduction

The course of biochemical and physiological actions results in the production of harmful free radicals and reactive oxygen species in human body. These free radicals and reactive oxygen species damage important biomolecules such as DNA, lipids, and proteins; ultimately becoming the leading source of different serious chronic disorders like cancer, aging, diabetes, atherosclerosis and several neurodegenerative disorders (1). To overcome this hazard, nature provides us a defense shield in the form of dietary antioxidants from plants (2). Different synthetic antioxidants are routinely used in medical practices, but may be unsafe because of their side effects and toxicity to off-target organs of concern. It remains possible that natural antioxidants derived from plant sources may be of vast use for human consumption and nutritional supplementation. 

In this regard, *Geranium* (cranesbill) species are well known for their polyphenolic contents and traditional usage. Different *Geranium* species (Geraniaceae) have been employed in folk medicine for their tonic, antidiabetic, antidiarrheal, antihemorrhoidal, and diuretic properties. Furthermore, they have been used in the treatment of cough, whooping cough, fever, tonsillitis, urticaria, dysentery, kidney pain and gastrointestinal diseases worldwide (3, 4). The leaves of some *Geranium* species are consumed as food in traditional Turkish cuisine such as salad or added to pastry (3, 5, 6) *Geranium* species have been shown to possess antiprotozoal, α-glucosidase and HIV reverse transcriptase inhibitory, antioxidant, antibacterial, and antiviral activity against influenza A and B viruses (7-12). Due to their diverse therapeutic benefits and traditional usage, it is interesting to research *Geranium* species from biological and phytochemical point of view (3, 7, 13). The aim of the present study is to assess any possible radical scavenging and cytotoxic activities of different* Geranium *extracts which are widely used in traditional Turkish medicine in order to find promising herbal extracts and compounds for drug discovery. 

In this study, in vitro antiradical properties as well as cytotoxic activities of different extracts of *G. stepporum* and *G. psilostemon *were investigated. Their radical scavenging activities were tested against nitric oxide (NO) and superoxide (SO) radicals. In addition, trolox equivalent total antioxidant capacities (TEAC) of the extracts were determined using 2,2’-azino-bis (3-ethylbenzothiazoline-6-sulfonic acid) diammonium salt (ABTS). Their cytotoxic activities were investigated against KB human epidermoid carcinoma cell line based on cell proliferation assay. To clarify phytochemical contents of the extracts, their total phenolic, flavonoid, flavonol contents and their comparative HPLC profiles were investigated.

## Experimental

*Plant materials*

*Geranium psilostemon* Ledeb. was collected from Hamsiköy in Agust 2006, Trabzon and* Geranium stepporum* Davis was collected from Egrisogut, Pinarbasi, Kayseri in May 2006. The plants were identified by Prof. Dr. M. Koray Sakar, Hacettepe University, Ankara, Turkey. Voucher specimen was deposited in the Herbarium of the Faculty of Pharmacy, Hacettepe University, Ankara, Turkey (HUEF 06001, 06003).

*Preparation of the herbal extracts*

Dried and powdered aerial parts of the plants (20 g) were extracted with 80% methanol (MeOH) (4 × 50 mL) at room temperature and the combined MeOH extracts were concentrated under reduced pressure. The resultant extracts were dissolved in H_2_O and the water-soluble portion was partitioned against petroleum ether (40–60°C) (4 × 15 mL), EtOAc (6 × 15 mL) and *n*-butanol (*n*-BuOH) (4 × 15 mL), respectively. Extracts: *G. psilostemon*-H_2_O (1), *G. psilostemon*-*n*-BuOH (2), *G. psilostemon*-EtOAc (3), *G. stepporum*-H_2_O (4), *G. stepporum*-*n*-BuOH (5), *G. stepporum*-EtOAc (6).

*SO radical scavenging effect by alkaline DMSO method*

The method of Elizabeth and Rao was used for the detection of superoxide radical scavenging activities of the extracts with slight modification. Briefly, a superoxide radical was generated in a nonenzymatic system. The reaction mixture containing 10 µL of Nitro Blue Tetrazolium (NBT, 1 mg/mL solution in dimethylsulfoxide –DMSO-) and 30 µL of the extract or reference compound (25, 100, 200, 400 and 800 µg/mL) were dissolved in DMSO. 100 µL of alkaline DMSO (1 mLDMSO containing 5 mM NaOH in 0.1 mL water) was added to give a total volume of 140 µL and the absorbances were measured at 560 nm using a microplate reader (14, 15). Quercetin was used as positive control for this experiment. All tests were conducted in triplicate.

*NO scavenging* *effect*

In order to determine the NO radical scavenging activities of the extracts, 60 μL of serial dilutions of each sample (25, 100, 200, 400 and 800 µg/mL) were added into a 96-well, flat-bottomed plate. Following this, 60 μL of 10 mM sodium nitroprusside, dissolved in phosphate buffered saline (PBS), was added to each well and the plate was incubated under light at room temperature for 150 min. Finally, an equal volume of Griess reagent (1% sulfanilamide, 0.1% naphthylethylenediamine dihydrochloride, 2.5% H_3_PO_4_) was added to each well in order to measure the nitrite content. After 10 min, when the chromophore was formed, the absorbances at 577 nm were measured in a microplate reader (16, 17). Quercetin was used as positive control for this experiment. All tests were conducted in triplicate.

*TEAC (Trolox Equivalent Antioxidant Capacity) assay*

The method is based on the ability of antioxidant molecules to quench ABTS , a blue-green chromophore with characteristic absorption at 734 nm, in comparison with the antioxidant potency of Trolox, a water-soluble α-tocopherol analog. The addition of antioxidants to the preformed radical cation reduces ABTS  to ABTS, determined by a decolorization. ABTS  was generated by reacting ABTS (7.4 mM) with potassium persulphate (2.6 mM). The solution was diluted to obtain an absorbance of 1.4 units at 414 nm with ethanol at 734 nm. The ABTS  antioxidant reaction mixture contained 200 µL of ABTS  solution, and 50 µL of extract or water for control. The absorbances at 420 nm of the resulting solutions were measured at the 6^th^ minute of the reaction by a 96-well plate reader. Results are expressed as TEAC in µM of Trolox per 50µg/mL of extract (18). All tests were conducted in triplicate.

*Cytotoxic activity against KB cell line*

Cell suspension (0.1 mL) of KB human epidermoid carcinoma cell line was seeded into 96-multi-well plates at the concentration of 1x10^4^ cells/mL and cultured for 24 h. Cells were incubated with various concentrations of the test samples in a humidified 5% CO_2_ incubator at 37^o ^C for 48 h (10 – 0.1 µg/mL). After incubation, the cells were washed and placed in a fresh medium. 20 µL of MTT solution (3 mg/mL in PBS) was added to this medium and the cells were incubated for 4 h. After aspiration of the media, 100 µL of DMSO was added to each well to dissolve the formed formazan. The absorbances were measured at 577/655 nm using a microplate reader (19). Adriamycin was used as a positive control. The data points represent triplicate determinations from the representative experiment that produced similar results twice.

*Estimation of total phenolic content*

The total phenolic content was determined using Folin-Ciocalteu reagent. Briefly, 10 µL of sample or standard (10-100 µM catechin) plus 150 µL of diluted Folin- Ciocalteu reagent (1:4 reagent:water) was placed in each well of a 96-well plate and incubated at room temperature for 3 min. Following the addition of 50 µL of sodium carbonate (2:3 saturated sodium carbonate:water) to each well and a further incubation of 120 min at room temperature, the absorbances were read at 725 nm. Results are expressed as gallic acid equivalents (GAE) in milligrams per gram extract. All tests were conducted in triplicate (20).

*Determination of total flavonoids*

Total flavonoids were determined using the method based on the formation of a flavonoid-aluminum complex (21). 0.5 mL of 2% AlCl_3_ ethanol solution was mixed with 0.5 mL of the sample solution (1 mg/mL). The resultant mixture was incubated for 1 h at room temperature for yellow color development which indicated the presence of flavonoids. The absorbances were measured at 420 nm using UV-VIS spectrophotometer (Bio-Tek Instruments, M-Quant Biomolecular spectrophotometer). Total flavonoid content was calculated as quercetin equivalent (mg/g) using the equation obtained from the curve, y = 0.0187x + 0.2211, where x is the absorbance and y is the quercetin equivalent (mg/g). All tests were conducted in triplicate.

*Determination of total flavonols*

Total flavonol contents were determined using the method in the literature (22). Two milliliter of the plant sample (1 mg/mL) was mixed with 2 mL of 2% AlCl_3_ in ethanol and 3 mL of (50 g/l) sodium acetate solution. The mixture was incubated at 20 ºC for 150 min, and then the absorbances were measured at 440 nm. Total flavonol content was calculated as quercetin equivalent (mg/g) using the following equation based on the calibration curve, y = 0.0078 + 2.4957, where x is the absorbance and y is the quercetin equivalent (mg/g). All tests were conducted in triplicate.

*Phytochemical analyses of the extracts by* *HPLC*

HPLC investigations were performed on a Dionex HPLC instrument consisting of a P60 HPLC pump, Dionex ASI-100 autosampler, and Dionex Photodiod Array Detector. The separation was carried out on a Hichrom-Nucleosil 100-5 C_18_ column (5µm, 250mm X 4.6mm) under the following chromatographic conditions: sample injection volume, 10 μL; column temperature, 23°C; flow rate, 1.0 mL/min; mobile phase, acetonitrile and 0.1% aqueous phosphoric acid (v/v). A gradient program was used according to the following profile: 0–5 min, 2–10% acetonitrile; 5-10 min, 10–14% acetonitrile; 10-15 min, 14–18% acetonitrile; 15-18 min, 18%; 19-24 min, 18-22% acetonitrile; 25-36 min, 22-28%; 36-42 min, 28-80%; 42-52 min, %80. The wavelength of UV detection was set at 280 nm. Gallic acid, methyl gallate, and pusilagin, which were previously isolated from *G. lasiopus,* were used as standard compounds for HPLC studies (23).

*Statistical analyses*

The data were expressed as mean ± standard deviation (SD) for at least three independent determinations in triplicate or quadruplicated for each experimental point. Each experiment was repeated at least three times for each sample to establish concentration–response curve. The concentration at which the extent of oxidation was suppressed by 50% (IC_50_ µg/mL) was calculated from each concentration–response curve. The results obtained were statistically processed using Microsoft Excel program. Student’s *t*-test was applied and *p *< 0.05 was accepted for statistical significance. Percentages of inhibition achieved by different concentrations of the extracts were calculated by the following equation: [I(%)= (A_0_-A)/A_0_ x 100], where A_0_ is the absorbance of the control reaction and A is the absorbance of the examined samples corrected for the value of blank probe.

## Results and Discussion

*SO radical scavenging effect*

**Figure 1:**
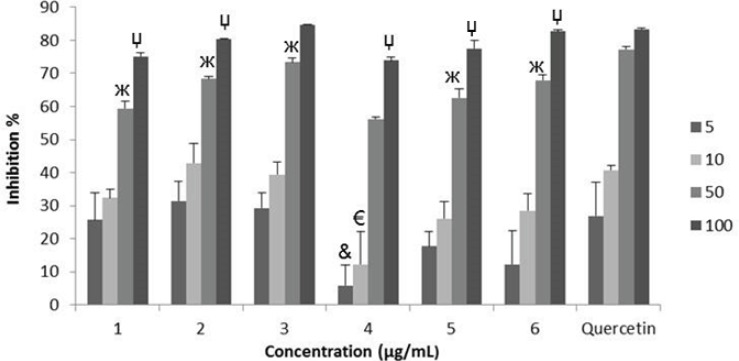
SO radical scavenging effects of the extracts: *G. psilostemon*-H_2_O (1), *G. psilostemon*-*n*-BuOH (2), *G. psilostemon*-EtOAc (3), *G. stepporum*-H_2_O (4), *G. stepporum*-*n*-BuOH (5), *G. stepporum*-EtOAc (6). ^&^p<0.05: Significantly different from quercetin 5 µg/mL, ^€^p<0.05: Significantly different from quercetin 10 µg/mL, ^ж^p<0.05: Significantly different from quercetin 50 µg/mL, ^џ^p<0.05: Significantly different from quercetin 100 µg/mL, Results are expressed as mean SD values of three observations

Superoxide radical is considered as a major biological source of reactive oxygen species (24). Although superoxide anion is a weak oxidant, it gives rise to the generation of powerful and dangerous hydroxyl radicals as well as singlet oxygen, both of which contribute to oxidative stress (25). The superoxide radical scavenging activities of different extracts of *Geranium* species were compared with the same doses of quercetin ranging from 5-100 μg/mL. They showed dose dependent SO radical scavenging activities which are very close to those of quercetin (Figure 1). On the other hand, n-BuOH (2) and EtOAC (3) extracts of *G. psilostemon* were found to have the highest SO radical scavenging activity (IC_50_ 29.4 µg/mL) compared with the other extracts. 

**Figure 2:**
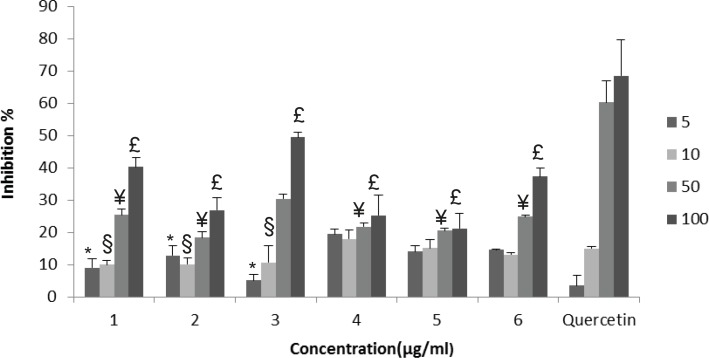
NO radical scavenging effects of the extracts: *G. psilostemon*-H_2_O (1), *G. psilostemon*-*n*-BuOH (2), *G. psilostemon*-EtOAc (3), *G. stepporum*-H_2_O (4), *G. stepporum*-*n*-BuOH (5), *G. stepporum*-EtOAc (6). * p<0.05: Significantly different from quercetin 5 µg/mL, § p<0.05: Significantly different from quercetin 10 µg/mL, ¥ p<0.05: Significantly different from quercetin 50 µg/mL, £ p<0.05: Significantly different from quercetin 100 µg/mL, Results are expressed as mean SD values of three observations

**NO scavenging effect**

Nitric oxide is a very unstable oxygen metabolite under aerobic conditions. It reacts with O_2_ to produce its stable product nitrate and nitrite through intermediates NO_2_, N_2_O_4,_ and N_3_O_4_. NO scavenging effects of the extracts were determined using Griess reagent. Tested extracts showed dose dependent NO scavenging activities, however they were found significantly lower than that of quercetin. EtOAc extract of *G. psilostemon* (3) was found to have the highest NO radical scavenging activity (IC_50_ 98.4 µg/mL) compared with the other extracts (Figure 2). 

*TEAC (Trolox equivalent antioxidant capacity) assay*

**Table 1:** Antioxidant capacities of the extracts determined by TEAC method.*G. psilostemon*-H_2_O (1), *G. psilostemon*-*n*-BuOH (2), *G. psilostemon*-EtOAc (3), *G. stepporum*-H_2_O (4), *G. stepporum*-*n*-BuOH (5), *G. stepporum*-EtOAc (6). Results are expressed as mean SD values of three observations

**Extract (100 µg/mL)**	**TEAC (µM TE)**
1	0.284 ± 0.07
2	0.301 ± 0.30
3	0.371 ± 0.29
4	0.262 ± 0.34
5	0.300 ± 0.21
6	0326 ± 0.28

ABTS• radical scavenging activities of the extracts were determined and both EtOAc extracts of *G. stepporum* and *G. psilostemon* showed the highest activity and their TEAC values for 50 µg/mL concentration were found as follows: 0.326 ± 0.28 and 0.371 ± 0.29 µM of Trolox, respectively. For the other tested extracts, TEAC values were found higher for *n*-BuOH extracts than those of the water extracts of both species (Table 1).

**Figure 3:**
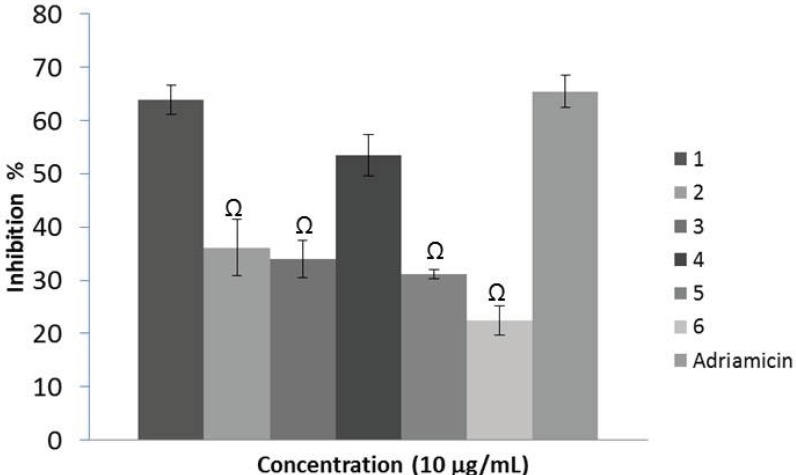
Effects of the extracts (10 µg/mL) on the viability of *KB cell line* after 48 h. *G. psilostemon*-H_2_O (1), *G. psilostemon*-*n*-BuOH (2), *G. psilostemon*-EtOAc (3), *G. stepporum*-H_2_O (4), *G. stepporum*-*n*-BuOH (5), *G. stepporum*-EtOAc (6). ^Ω^ p<0.05: Significantly different from Adriamicin. Results are expressed as mean ±SD values of triplicate determinations from the representative experiment that produced similar results twice. Adriamicin was used as a positive control and at 0.01 µg/mL concentration

*Cytotoxic activity against KB cell line*

Cytotoxicity activities of the extracts were evaluated using microculture assay based on the metabolic reduction of MTT in the concentration range of 0.1 – 10 µg/mL. This technique permitted to evaluate the dose-dependent effects by linear regression analysis showing acceptable R^2^ values and correlation. The results of the cytotoxic activity assay showed that all the tested extracts showed dose-dependent but negligible cytotoxicity against KB cell line below 10 µg/mL concentration (Figure 3). Both *n*-BuOH and EtOAc extracts of two species showed inhibition lower than 30% on the viability at 10 µg/mL. However, water extracts of *G*. *psilostemon* and *G. stepporum *inhibited more than 50% of the proliferation at the same concentration. This result indicated that while strong radical scavenging activities were observed for *n*-BuOH and EtOAc extracts, cytotoxicity was found only for the water extracts of the species.

*Total phenolic content*

In this study, total phenolic contents of the extracts of two different *Geranium* species were expressed as gallic acid equivalent in mg/g dry extract. [1 (224.6401±0.21), 2 (281.0769±0.23), 3 (345.0679±0.12), 4 (208.1012±0.82), 5 (271.885±0.42), 6 (389.0881±0.84)]. EtOAc extracts of both species (3 and 6) were found to possess the highest total phenolic content among the tested extracts. 

*Total flavonoids*

Total flavonoid contents were expressed as quercetin equivalent (mg/g): 1 (18.6697±0.21), 2 (114.5871±0.43), 3 (68.6697±0.32), 4 (7.7385±0.58), 5 (116.5779±0.13), 6 (7.7385±0.22). *n*-BuOH extracts of both species (2 and 5) had the highest flavonoid contents.

*Total flavonols*

Total flavonol contents were calculated as quercetin equivalent (mg/g): 1 (0), 2 (26.5769±0.32), 3 (7.8974±0.21), 4 (0), 5 (58.6666±0.48), 6 (3.7620±0.36). *n*-BuOH extracts of both species (2 and 5) had the highest flavonol contents.

**Figure 4:**
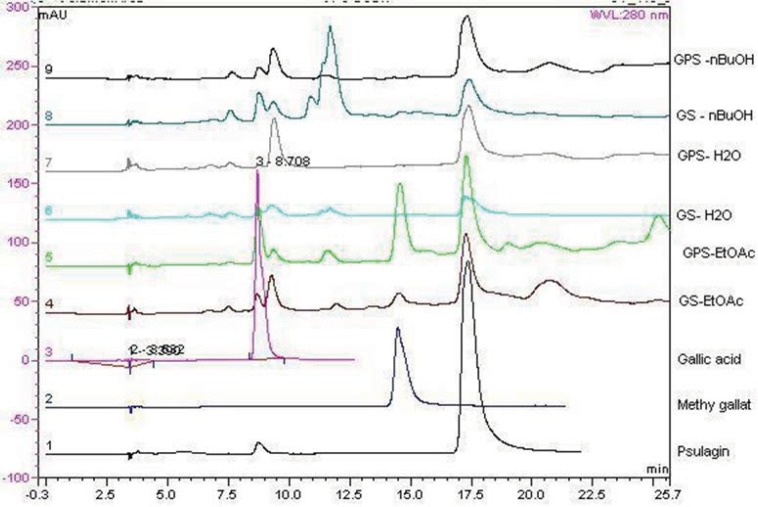
HPLC chromatograms of the extracts

*HPLC analyses of the extracts*

**Figure 5:**
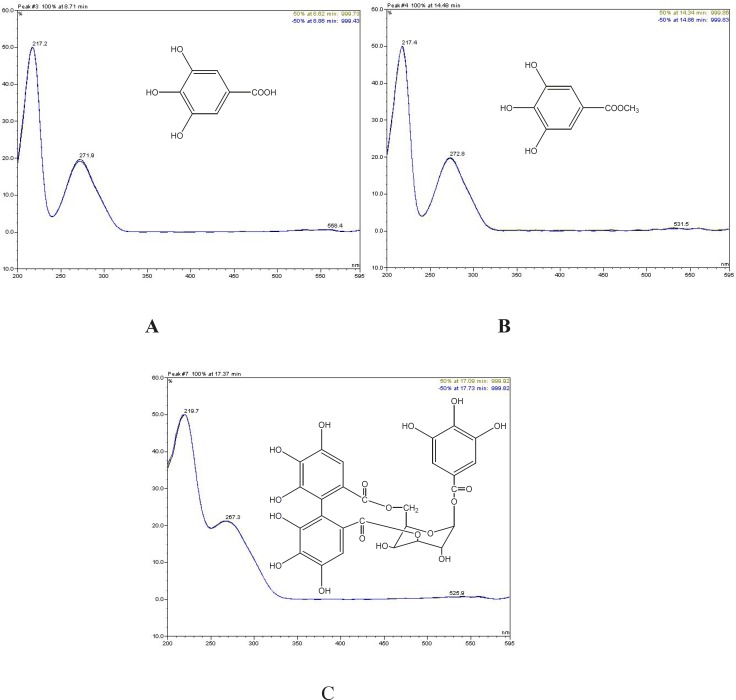
Structures and UV spectra of A: gallic acid, B: methyl gallat, C: pusilagin

Chromatograms of the tested extracts and the standard compounds are shown in Figure 4. In order to find out which polyphenols are responsible for the observed antioxidant activity of the extracts, HPLC analyses* were applied to Geranium* extracts and the standard compounds. Gallic acid, methyl gallate, and pusilagin, which were previously isolated from *G. lasiopus*, were used as standard compounds (Figure 5). Their UV spectra and retention times were used for the evaluation of the extracts (Figure 4)

Comparison of *G. psilostemon*-H_2_O (1), *G. psilostemon*-*n*-BuOH (2), *G. psilostemon*-EtOAc (3), *G. stepporum*-H_2_O (4), *G. stepporum*-*n*-BuOH (5) and *G. stepporum*-EtOAc (6) with standard compounds showed that both EtOAc extracts contained gallic acid, methyl gallate and pusilagin; both *n*-BuOH extracts contained gallic acid and pusilagin; and both water extracts contained pusilagin as a major gallic acid derivatives. According to the results above, pusilagin is one of the major compounds in all extracts. This result indicates that three gallic acid derivatives, gallic acid, methyl gallate and pusilagin, are the important compounds for the bioactivities of *Geranium* species. 

Free radicals are known to play a definite role in a wide variety of pathological conditions. Antioxidant foods and compounds fight against free radicals and protect us from various diseases. They exert their actions either by scavenging the reactive oxygen species or protecting the antioxidant defense mechanisms (26). Plant materials rich in phenolics are increasingly being used in the food industry since they retard oxidative degradation of lipids and improve the quality and nutritional value of food (27). Phenolic compounds of plants are also very important because their hydroxyl groups confer scavenging ability comparable with the findings in the literature for other extracts of plant products (28). Our results suggest that gallic acid derivatives and flavonoids may be the major contributors for the antioxidant activity since the IC_50_ values of radical scavenging activities of various tested *Geranium* extracts and the contents of phenolics or flavonoids exhibited correlation. 

The involvement of free radical mediated cell damage in many different diseases has led us to determine antioxidant and cytotoxic activities of the *Geranium* species which are medicinal food plants. Concerning the above results, phytochemical contents and bioactivities of *G. psilostemon* and *G.stepporum* were found to be very similar to each other. While all the tested extracts showed dose dependent bioactivity, *n*-BuOH extract (2) and EtOAC extract (3) of *G. psilostemon* were found to be the most effective extracts in SO radical scavenging activity tests. EtOAc extracts of *G. psilostemon* (3) were the most effective NO radical scavengers and had the highest TEAC value. Total phenolic contents were found to be the highest for the EtOAc extracts, while total flavonoid and flavonol contents were found to be the highest for the *n*-BuOH extracts. According to the results above, strong radical scavenging activities of the EtOAc extracts may be coming from the presence of gallic acid derivatives in both extracts. In addition, it is noteworthy that while EtOAc and *n*-BuOH extracts of both species demonstrated higher radical scavenging activities and higher phenolic contents, these extracts did not show cytotoxicity at the tested concentrations. However water extracts of both species showed cytotoxicity at 10 µg/mL. These results indicate that, pusilagin, which is a major compound of water extracts, is important for the cytotoxic activities of the water extracts together with the nonphenolic compounds as gallic acid and methyl gallate, which are simple phenolics, stand out for the radical scavenging activities of the tested extracts.

Polyphenolic compounds have been shown to possess significant antioxidant activities, which could be due to their ability to absorb, neutralize, and quench free radicals (29). Strong radical scavenging activities of extracts could be based on the presence of hydroxyl groups attached to the aromatic ring structures, which help to quench radicals (30). Antioxidant activity is due to specific polyphenols present in extracts, or apart from polyphenols, antioxidant activity is also due to other phytochemical compounds, and/or there is a synergism between polyphenols and other phytochemical compounds. For example a number of studies have shown that plant polyphenols, when combined with each other or with other antioxidants, exhibit stronger antioxidant activity compared to their individual activity (31). 

## Conclusions

Natural antioxidants are useful in food industry as preservatives as they increase the shelf life of food products by preventing the loss of their sensory and nutritional quality. The replacement of synthetic antioxidants such as butylhydroxyanisole (BHA) and butylhydroxytoluene (BHT) with natural ones is a desirable aim since the former have been linked to carcinogenesis (32). Therefore, consumption of antioxidant food products or herbal extracts is considered to have a beneficial effect on human health. According to our results, *G. psilostemon* and *G. stepporum* could be considered good sources of natural antioxidants for application in food industry because these species are rich sources of phenolic constituents, Additional investigations are in progress for the future development of antioxidants from *Geranium* species by different mechanisms. 
